# Ionic transport kinetics and enhanced energy storage in the electrode/poly(*N*-vinyl imidazole) interface for micro-supercapacitors†

**DOI:** 10.1039/d0ra08137j

**Published:** 2020-12-21

**Authors:** Karthik Krishnan, Selvakumar Karuthapandi, Saranyan Vijayaraghavan

**Affiliations:** Corrosion and Material Protection Division, CSIR- Central Electrochemical Research Institute (CECRI) Karaikudi TN 630-003 India; Department of Chemistry, School of Science and Languages, VIT-AP University Amaravati AP 522-237 India karthikk@cecri.res.in selvakumar.k@vitap.ac.in

## Abstract

The detailed understanding and control of ionic transport pathways in the electrode/electrolyte interface is vital for realizing micro-scale energy storage devices and formulating adequate design principles. A planar device geometry with nanostructured thin solid polymer electrolyte (SPE) and potassium hydroxide (KOH) incorporated poly(*N*-vinyl imidazole) (PVI) is demonstrated for micro-supercapacitors (MSCs). The adsorption/desorption kinetics of ionic charges in the interfacial regime of ITO/PVI–KOH has been investigated for electrical double layer capacitance (EDLC) characteristics. A single-cell of ITO/PVI–KOH/ITO planar MSC shows the large variation in volumetric capacitance and capacitance retention characteristics when the thickness of PVI–KOH approaches the characteristic nanoscale. Moreover, ITO/PVI–KOH/ITO planar MSC consisting of five series-cells exhibits the maximum operating cell voltage of 5.0 V with maximum volumetric energy and power density of 0.056 mW h cm^−3^ and 6.89 mW cm^−3^, respectively. The electrochemical properties of planar MSC have been systematically studied so as to confirm how the anions and cations are separated at electrode/electrolyte interfaces by means of an electromotive force. Significantly, the hydrated PVI enables charge migration and separation of cations and anions at the electrode/electrolyte interfaces.

## Abstract 2

The ionic charge transport kinetics in the poly(*N*-vinylimidazole) and energy storage at the electrode/poly(*N*-vinylimidazole) interfaces have been systematically studied for high performance micro-supercapacitors.

## Graphical abstract

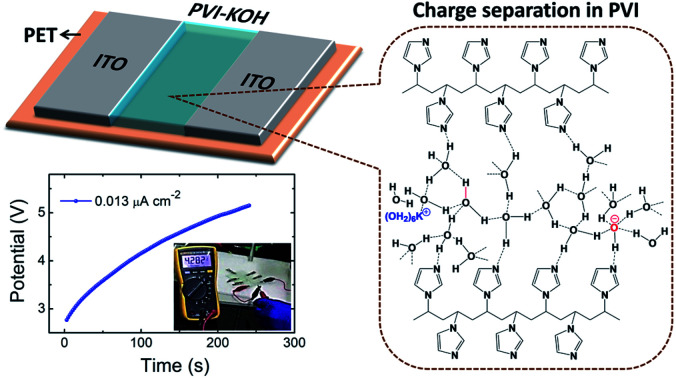

## Introduction

With increasing demand for wearable and portable electronic devices, micro/nanoscale power systems have received intense attention due to their ultra-high power density and high rate capability.^1–5^ Miniaturization and integration of micro-power systems such as micro-supercapacitors (MSCs), micro-batteries and piezoelectric energy converters with on-chip devices offer great potential for future portable electronics.^6–8^ Among various power systems, MSCs have attracted considerable attention because of their high capacitance, high energy density and large endurance characteristics.^8–11^ In MSCs, electrical energy is stored at the electrode/electrolyte interface either by the reversible adsorption/desorption of charges (non-faradaic) or rapid redox reaction of charges (faradaic).^12,13^

Till now, many reports available to elucidate the high performance supercapacitance characteristics by specifically modifying the properties of electrode materials.^14–18^ But, the supercapacitance characteristic not only depends on the electrode materials, but also relies on electrolyte properties and new device architecture.^19–21^ In recent years, structurally confined solid polymer electrolyte (SPE) offers many advantages in fabricating MSCs without modifying the properties of electrode.^22–24^ Confinement of SPE film to the characteristic nanoscale can reveal variation of inter-polymer chain interaction, which influences the ionic conductivity and stability characteristics. Furthermore, supercapacitance characteristics become more pronounced when the SPE film confined to certain nanoscale due to the synergistic effect of shorter ionic-diffusion pathways and low equivalent series resistance (ESR).^25–28^

Herein, we demonstrated a poly(*N*-vinyl imidazole) (PVI)-based planar MSCs on indium tin oxide (ITO) coated flexible PET substrate through a micro-edging of ITO films. A planar device architecture consists of thin ITO electrodes separated by potassium hydroxide (KOH) salt-incorporated PVI electrolytic film (ITO/PVI–KOH/ITO) has been fabricated. In order to understand the interfacial interaction between imidazole group of PVI and KOH for supercapacitance characteristics, planar MSCs were fabricated with various PVI–KOH film thicknesses. PVI–KOH with thicker film shows higher ionic conductivity that led to the higher volumetric capacitance of planar MSC. In contrast, the reduction of PVI–KOH film thickness decreases the ionic conductivity as well as the volumetric capacitance. Consequently, a single cell of ITO/PVI–KOH/ITO planar MSC with ∼121 nm of PVI–KOH reveals higher volumetric capacitance (128 mF cm^−3^) and 92% capacitance retention less than 1400 continuous cycles. But, the reduction of PVI–KOH film thickness to the characteristic nanoscale (∼28 nm) remarkably enhances the capacitance retention (∼95%) for more than 3500 continuous cycles. This significant deviation in capacitance characteristic between thick and ultra-thin PVI–KOH films has attracted and enabled us to investigate the effect of polymer film thickness on EDLC formation mechanism and stability characteristics. To quantitatively analyse the performance characteristics of EDLC, planar MSC consisting of five series-cells also fabricated. Such multi-cell planar MSC exhibits extended operation voltage (5.0 V) with maximum volumetric energy and power density of 0.056 mW h cm^−3^ and 6.89 mW cm^−3^, respectively. These results explain the importance of confinement effect on solid polymer electrolyte for supercapacitance characteristics.

## Results and discussion

### Structural and morphological characteristics of prepared PVI–KOH complex

**Fig. 1:**
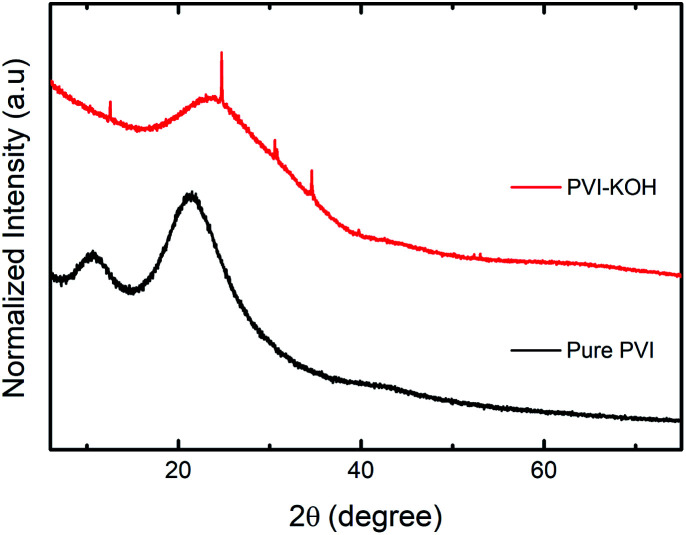
XRD patterns (normalized) of prepared pure PVI and PVI–KOH films with a thickness of ∼121 nm.

The structural investigations of the prepared pure PVI and KOH incorporated PVI thin films were carried out using various techniques. Fig. 1 shows the X-ray diffraction (XRD) patterns of both pure PVI and PVI–KOH thin films. The two major diffraction peaks found at 10.6° and 21.28° (2*θ*) correspond to the PVI polymer.^29^ By taking into account of the normalized peak intensity, two distinct peak in the pure PVI may be due to existence of crystalline ordering.^29,30^ In PVI–KOH thin film, the large variation in the peak profile is observed. Especially, four additional peaks are found in PVI–KOH film that mostly arise when the KOH loading exceeds the threshold limit.^31^ As compared to the pure PVI film, the decrease in peak intensity with large peak broadening mainly due to the amorphous nature of PVI–KOH film.

**Fig. 2:**
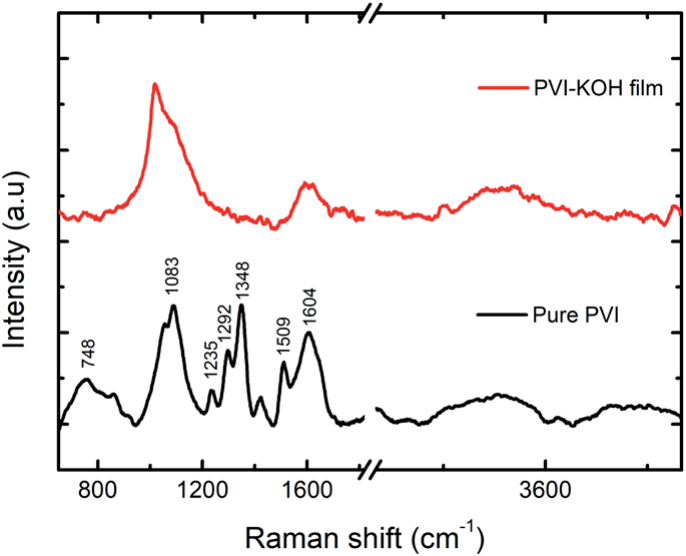
Raman spectra of prepared pure PVI and PVI–KOH films (∼121 nm).

To further understand the structural characteristics of the prepared PVI and PVI–KOH films, the Raman spectroscopy was conducted (Fig. 2). In the pure PVI film, the observed peak at 748 cm^−1^ corresponding to the stretching vibration of C–N bond that connects the imidazole group to the vinyl moiety and the backbone of the aliphatic chain. The major peak at 1083 cm^−1^ is attributed to the C–C–C bending vibration of the aliphatic chain. The C–H bending with C=N stretching of imidazole group showed a peak at 1235 cm^−1^. The peak at 1292 cm^−1^ arises due to the stretching of chain and ring breath. The peaks at 1348 and 1509 cm^−1^ and 1604 cm^−1^ can be because of the stretching of ring, combined stretching vibrations of C–C and C=N bond and characteristic C=C stretching vibration, respectively.^32^ But, in the PVI–KOH film, large peak broadening that cause disappearance of some normal modes, which reveals the amorphous nature of the film.^33,34^ It also suggests that the incorporation of large concentration of KOH in the PVI polymer significantly affects the PVI polymer structure.^31^

**Fig. 3:**
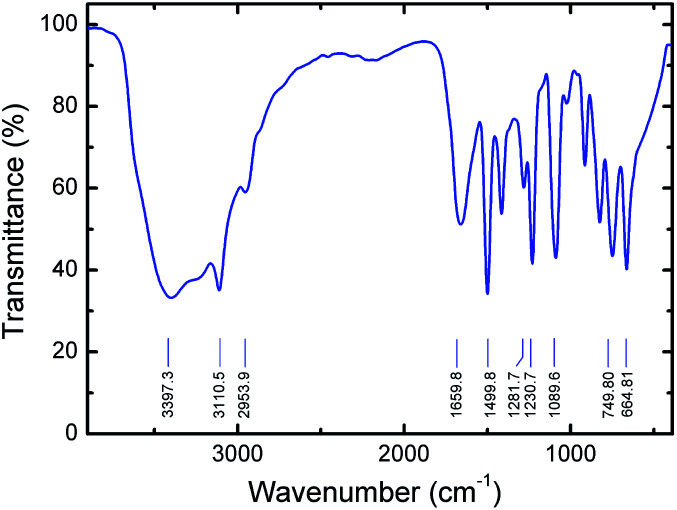
FTIR spectrum of prepared pure PVI.

Fourier transform infrared (FTIR) study was also performed on a prepared pure PVI in order to understand the vibrational properties of polymer under ambient condition. From FTIR spectra, the C–H and N–H stretching vibrations of PVI are observed, as shown in Fig. 3. The broad peak at ∼3397 cm^−1^ arises by the presence of bound water molecules in the PVI. The peak at ∼3110 cm^−1^ corresponding to C–H vibrations of the imidazole ring. The observed peak at ∼2953 cm^−1^ is due to the C–H stretching vibration of the polymer backbone, suggesting the polymer formation. The peak at ∼1659 cm^−1^ is ascribed to the characteristic of C=C stretching vibration of PVI. There are three major vibrational modes (∼1499, ∼1281, and ∼1230 cm^−1^) corresponding to the imidazole group of PVI. The stronger peak at ∼1089 cm^−1^ is assigned to the in-plane bending of the azole C–H group. Emergence of peaks at ∼914, ∼825 and ∼747 cm^−1^ are majorly attributed to the bending vibrations of heterocycles of PVI. The characteristic peak found at ∼664 cm^−1^ is corresponding to the imidazole ring-puckering vibration.^32^ These results suggest that the FTIR studies are in good agreement with the Raman spectroscopy.

**Fig. 4:**
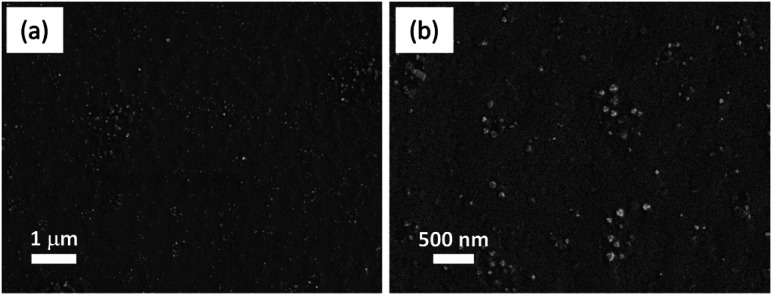
(a) FESEM images of PVI–KOH thin film (∼121 nm) used for ITO/PVI–KOH/ITO planar MSC, and (b) corresponding magnified view.

The topography of the microscopic analysis of PVI–KOH thin films were analyzed using both field-emission scanning electron microscopy and scanning probe microscopy. Fig. 4 shows the FESEM images of the PVI–KOH thin film (∼121 nm) measured in the ITO/PVI–KOH/ITO planar MSC device. It is clearly seen that the KOH salt is distributed randomly throughout the surface of the film. However, the PVI–KOH showed smooth surface with large-scale polymer alignment pattern (Fig. 4a). Fig. S1a–c in ESI† shows the AFM images of PVI–KOH planar MSC device with various thicknesses. All the films exhibit smooth surface with roughness at nanoscale. It was observed that the surface roughness of the film slightly increases with an increase in film thickness, which mostly arises due to the distribution of KOH salt in the matrix polymer. Fig. S1d (ESI†) represents the measurement technique adopted to find the thickness of the thin PVI–KOH film.

### Electrochemical performance of PVI-based planar MSC

**Fig. 5:**
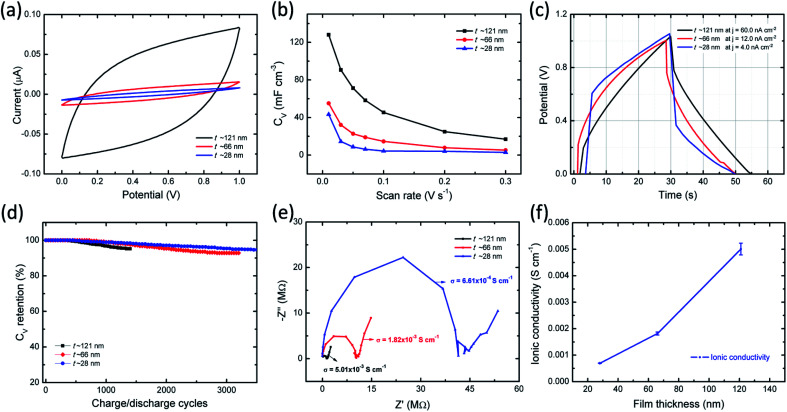
EDLC characteristics of ITO/PVI–KOH/ITO planar MSC as function of film thickness; (a) CV curves with a constant scan rate of 50 mV s^−1^. (b) Scan rate dependent volumetric capacitance (*C*_V_) plots. (c) Equivalent charging-discharging effect of planar MSCs with different current densities (*j*). (d) Capacitance retention as function of charge–discharge cycles. (e) Nyquist plots of planar MSC. (f) Film Thickness dependent ionic conductivity (error bar is estimated by three different planar MSCs with similar device geometry).

Electrochemical properties of ITO/PVI–KOH/ITO planar MSCs were investigated using two-electrode cell configuration. Cyclic voltammetry (CV) studies were performed on a single-cell planar MSC with various scan rates (10–300 mV s^−1^) and constant potential window of 0–1 V. To understand the interfacial interaction between polymer chains (PVI) and metal salts (KOH) on EDLC characteristics, PVI–KOH film thicknesses were varied in the planar MSC device configuration. Fig. 5a represents the CV curves of the planar MSC measured at constant scan rate (50 mV s^−1^) with various PVI–KOH thicknesses ranging between ∼28 nm and ∼121 nm. The CV plots of thickness dependent planar MSC exhibited a quasi-rectangular behavior, indicating the formation of an electrical double layer at electrode/electrolyte interfaces. It is obvious that the characteristic CV curve of planar MSC decreases with the decrease of film thickness. Also, the scan rate dependent CV measurement was conducted to evaluate the thickness dependent planar MSC on supercapacitance characteristics. Fig. S2 (ESI†) shows the CV curves of the thickness dependent planar MSCs as function of scan rates. The CV curve retained their quasi-rectangular profile in all the scan rates, which demonstrates that the PVI-based planar MSCs have an ideal capacitance characteristics and good rate capability. The volumetric capacitance (*C*_V_) of planar MSC was estimated by taking into account of CV plot. Fig. 5b shows the estimated *C*_V_ curves of thickness dependent planar MSC. Significantly, the planar MSC with a PVI–KOH thickness of ∼121 nm revealed higher *C*_V_ of 128 mF cm^−3^ at 10 mV s^−1^. The decrease of PVI–KOH thickness to ∼28 nm showed drop of CV to 43 mF cm^−3^ at 10 mV s^−1^. Furthermore, the *C*_V_ decreased with an increase in scan rates in all the cases and exhibited a minimum value of 2.9 mF cm^−3^ at 300 mV s^−1^ for a PVI–KOH thickness of ∼28 nm.

Galvanostatic charge–discharge (GCD) behavior of planar MSC was investigated as function of PVI–KOH thickness. Fig. 5c represents the GCD plots of thickness dependent planar MSCs with various current densities, which is essential to estimate the charge–discharge characteristics at specified time constant. The charge–discharge rate at around 50 s is observed for all the planar MSCs. Also, the current density decreases with decrease in PVI–KOH thickness in order to obtain similar charge–discharge rate. Fig. S3 of ESI† shows the GCD plots of thickness dependent planar MSCs with various current densities. The single-cell of thickness dependent planar MSC exhibited a GCD pattern close to triangular shape at all current densities, suggesting an effective electrical double layer formation and high charge propagation across the electrodes.

To evaluate the cycling characteristics of the fabricated planar MSC device, the GCD studies were carried out according to the device capability. In Fig. 5d, the planar MSC device with higher PVI–KOH film thickness (∼121 nm) exhibited lower cycling performance of ≤ 1400 continuous cycles and ∼92% capacitance retention. It was observed that, both the cycling performance and capacitance retention increases with the reduction of PVI–KOH thickness to certain characteristic nanoscale. The planar MSC with ultra-thin PVI–KOH (∼28 nm) showed higher cycling performance of ≥3500 continuous cycles and large capacitance retention (∼95%), as shown in Fig. 5d.

To understand the ionic transport characteristics of the planar MSC device, the electrochemical impedance spectroscopy (EIS) was conducted. The Nyquist plot shows (Fig. 5e) a semicircle with vertical response in the low frequency region, which corresponds to the ideal capacitance behavior. The Nyquist plot was further analysed by fitting with equivalent circuit model, as shown in Fig. S4 of ESI.† From Nyquist plot, the intercept in the real axis is modelled by a series resistor (*R*_S_), and the semicircle is modelled by a constant phase element (CPE_2_) with a parallel resistor (*R*_p_). The vertical response is assigned to be CPE_1_. The obtained standard error was as low as 7% for the fitting parameters. Each CPE is characterized by capacitance *C*_1_ and *C*_2_, respectively. Here, a single CPE, designated as CPE_1_ is due to the electrode polarization, exhibiting vertical response at low frequency region. Presence of large number of ions in the PVI interface that are easily attracted by the nonblocking ITO electrode, resulting in the formation of EDL. Furthermore, the impedance response in the medium frequency range is modelled by an *R*_p_/CPE_2_, which corresponds to the bulk behavior of the PVI–KOH electrolyte. The ionic conductivity of the planar MSC is estimated to be between 10^−3^ and 10^−4^ S cm^−1^ with various PVI–KOH thicknesses. To quantitatively analyze the ionic transport characteristics of planar MSC, three different devices were used in each thickness (error bar plot is shown in Fig. 5f).

**Fig. 6:**
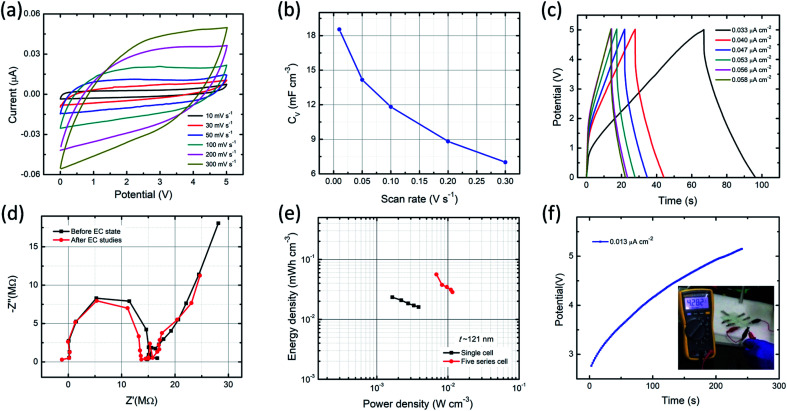
EDLC characteristics of five series-cells ITO/PVI–KOH/ITO planar MSC: (a) CV curves with various scan rates (b) Scan rate dependent volumetric capacitance (*C*_V_). (c) GCD profiles of the device at higher operating cell voltage of 5.0 V with different current densities. (d) Nyquist plots of the device under both pristine state and after all electrochemical studies. (e) Ragone plot comparison between single-cell and five series-cells planar MSC. (f) Charging behavior of device till 5.0 V with current density of 0.013 μA cm^−2^; the inset shows the photograph of a multimeter as load during discharging condition.

In order to estimate the performance characteristics of MSC, the ITO/PVI–KOH/ITO device with five series cell configuration also fabricated. For this study, we used a relatively thick PVI–KOH electrolyte (∼121 nm) to fabricate the five series-cells because of its higher ionic conductivity and higher volumetric capacitance. Fig. 6a represents the CV plots of the device studied at higher cell voltage of 5.0 V with various scan rates (10–300 mV s^−1^). It is obvious that the CV plot exhibits quasi-rectangular behavior in all the scan rates even at higher cell voltage, demonstrating an ideal EDLC characteristic with high rate capability. Moreover, the maximum volumetric capacitance of the five series cell planar MSC was found to be 18.5 mF cm^−3^ at low scan rate (10 mV s^−1^). The device exhibits decrease in volumetric capacitance with an increase in scan rate, as shown in Fig. 6b. The charge–discharge behavior of five series cell planar MSC was studied at higher cell voltage (5.0 V) with various current densities, as shown in Fig. 6c. Significantly, the device exhibits typical charge–discharge pattern in all the current densities. It infers that the PVI–KOH based planar MSC can adopt to work in a wide potential window for practical applications. The Nyquist plots (Fig. 6d) show the ionic conductivity of five series cell planar MSC, which remains nearly same after all the electrochemical measurements relative to that of the pristine device.

The performance of the planar MSC was further demonstrated using Ragone plot, as shown in Fig. 6e. Ragone plot is essential to understand the relationship between energy density (*E*_V_) and power density (*P*_V_) by GCD profile. The PVI–KOH based single-cell of planar MSC shows maximum volumetric energy and power density of 0.023 mW h cm^−3^ and 1.64 mW cm^−3^, respectively. But, the five-cell planar MSC exhibited the maximum volumetric energy and power density of 0.056 mW h cm^−3^ and 6.89 mW cm^−3^, respectively. It infers that the PVI–KOH electrolyte confined to certain nanoscale can give excellent device characteristics, which are comparable to the previously available MSC devices (Table S1†). To confirm further on the supercapacitance characteristics, five-cell planar MSC was connected with multimeter as load and estimated the discharging effect after one complete charging (∼5.0 V). Fig. 6f shows the galvanostatic charging and discharging profiles (inset represents the corresponding photograph of multimeter display) of the planar MSC. It illustrates that the planar MSC discharges certainly after one complete charging (the corresponding real-time movie is shown in Movie S1, ESI†). This characteristic phenomenon of the ITO/PVI–KOH/ITO planar MSC comprising a nanostructured film revealed excellent EDLC characteristics, which promise for their use in practical applications.

**Fig. 7:**
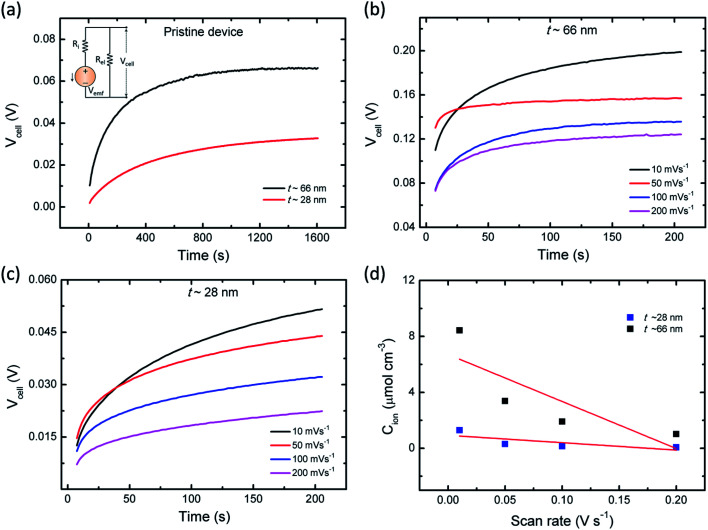
(a) The cell voltage (*V*_cell_) as function of time under pristine state (without applying a cyclic sweep; the inset represents the equivalent circuit model in emf generation). Time-dependent *V*_cell_ of the device measured with various cyclic sweep rates in (b) ∼66 nm (c) ∼21 nm thin PVI–KOH based planar MSC, respectively, and (d) linear plots of the scan rate dependent ionic concentration (*C*_ion_) under various PVI–KOH film thicknesses.

It is speculated that the effect of polymer confinement on the ionic transport at electrode/electrolyte interface has strong impact on the EDLC characteristics. The inclusion of ionic charges separated at the respective interfaces, in which the K^+^ ions at the negative ITO/PVI interface and OH^−^ ions at the positive ITO/PVI interface, gives rise to the generation of emf voltage (*V*_emf_) within the device.^35^ The inset of Fig. 7a shows the equivalent circuit model of the planar MSC under emf generation. Owing to the partial electronic component of the device, the theoretical *V*_emf_ cannot directly measured. By taking into consideration of the ionic resistance *R*_i_, and electronic resistance *R*_el_, the *V*_cell_ can be obtained from the following relation,^35,36^1
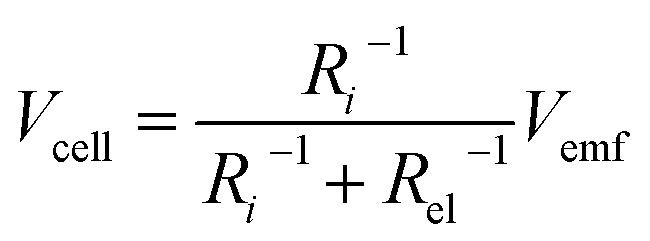
The *R*_el_ of the device is very high (>10^6^ Ω) due to the insulating nature of PVI–KOH. As consequence, the behavior of *V*_cell_ becomes closely associated with the *V*_emf_. In EDLC, the formation of *V*_emf_ can be described by the contribution of Nernst potential *V*_N_ and a diffusion potential *V*_d_. Hence, the total *V*_emf_ in the EDL device consists of^36,37^2
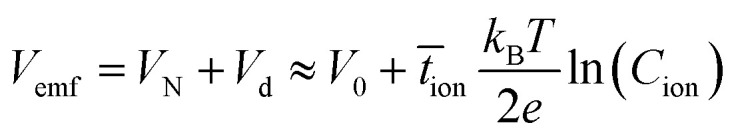
where *V*_0_ is the standard potential, *k*_B_ is Boltzmann constant, *e* is charge of electron, *T* is absolute temperature and *t̄*_ion_ is the mean ion transfer number (*t̄*_ion_ = *t̄*_K^+^_ + *t̄*_OH^−^_).^36^

Fig. 7a shows the time dependent *V*_cell_ of both thick (∼66 nm) and relatively thin (∼28 nm) PVI–KOH based planar MSCs at pristine state. Fig. 7b and c show the *V*_cell_ of the ∼66 nm and ∼28 nm PVI–KOH based planar MSC devices, measured under various scan rates (10 to 200 mV s^−1^). The measurement was carried out in two steps: initially, a positive cyclic sweep was performed as 0 V → 1 V → 0 V at certain sweep rate. Then, the *V*_cell_ was measured as a function of time, under an open circuit condition. In both cases, gradual increase in *V*_cell_ for more than 200 s was observed because of the equilibration of ionic charges (K^+^ and OH^−^) at the respective electrode/electrolyte interfaces. It was observed that the increase in scan rate decreases the *V*_cell_ for both cases; because, the ionic charge separated at the electrode/electrolyte interfaces is a rate limiting process. However, the relatively thick PVI–KOH (∼66 nm) based planar MSC showed higher *V*_cell_, which was almost one order of magnitude larger than that of the thin device. This can be attributed to the influence of ionic contribution at interfaces. To gain further insight into the ionic charge distribution in the interfaces, the mean ionic concentration (*C*_ion_) was calculated from cyclic sweep measurements. The increase in cyclic sweep rate changes the intensity and area of CV curve, suggests that the ion transfer controlled reaction may happen at the electrode/electrolyte interfaces. The difference in current density can be evaluated from the Randles–Sevcik equation,^38,39^3
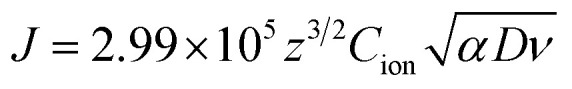
where *J* is the current density, *z* is number of electrons that assumed to be one, *C*_ion_ is the ionic concentration, *α* is the ion transfer coefficient,^38^*D* is the diffusion coefficient^39^ and *ν* is the scan rate. It is essential to mention that the concentrations of both K^+^ ions and OH^−^ ions are contributing equally to *C*_ion_ in eqn (3). Based on this assumption, *C*_ion_ was estimated from the cyclic scan and device geometry.^38,39^Fig. 7d represents the scan rate dependent *C*_ion_ for both thin and relatively thick PVI–KOH based MSC devices. It was found that the contribution of *C*_ion_ in thick planar MSC is higher than that of thin device. This can be attributed to the influence of ionic contribution at interfaces, resulting higher *V*_cell_ increases over time (Fig. 7b). Also, it is essential to mention that the cyclic scan rate can majorly influence the ionic contribution in EDLC. At small scan rate, large concentration of ionic species can be separated at the respective electrode/electrolyte interfaces due to the longer scan cycle. Whereas, the large scan rate significantly reduces the contribution of ionic carriers at electrode/electrolyte interfaces, resulting the decrease in *C*_ion_.

Based on these results, we infer the charge migration and separation in the PVI structure. The PVI has large number of imidazole pendant groups. Out of two nitrogen atoms in the imidazole ring, one nitrogen atom has a lone pair of electrons that are not contributing to the aromaticity. Consequently, that lone pair of electrons are available for hydrogen bonding formation and metal ion coordination. Since the PVI itself does not have any hydrogen bond donor, therefore, it has an inherent tendency to associate with large amount of water molecules through hydrogen bonding (Fig. S5, ESI†), which results PVI as hygroscopic in nature.

**Fig. 8:**
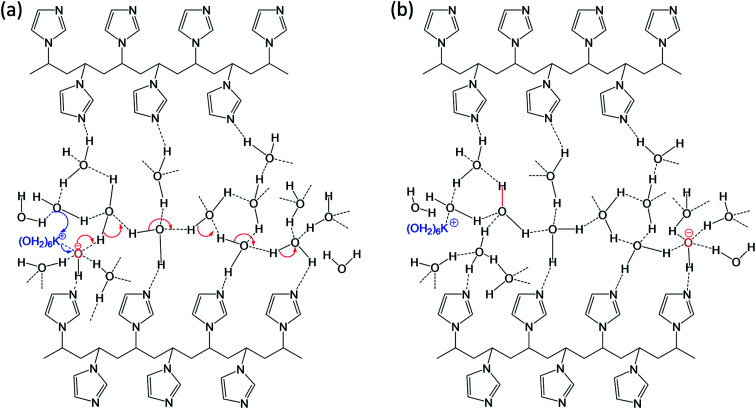
Proposed ionic transportation in the interfacial PVI structure under (a) pristine state and (b) charge separated state (biasing condition).

Our FTIR data also supports well with observation of broad OH stretching signal at ∼3397 cm^−1^ (*vide supra*) due to the PVI bound water molecules. Consequently, it is anticipated that the PVI bound water molecules in PVI–KOH film facilitates OH^−^⋯OH_2_ hydrogen bond and K^+^⋯OH_2_ coordination bond mediated ion transport. This assertion was further validated by RH dependent impedance spectroscopy studies. As shown in Fig. S6 of ESI,† the variation of environmental humidity around the PVI–KOH film significantly alters the *R*_ct_ values. It is apparent from Fig. S6,† higher relative humidity level lowers the *R*_ct_ implying the incorporation of more number of water molecules in PVI–KOH film under humid condition that in turn allows facile ion transport. The plausible mode of ion-transport is shown Fig. 8. According to the hard–soft acid–base (HSAB) theory, potassium ion is a hard Lewis acid that prefers to make coordination bond with hard Lewis base oxygen of water molecules than the relatively soft base nitrogen of imidazole ring. Not violating HSAB theory, in biology, while both N and O donors are prevalent in proteins, K^+^ is being transported across the membrane along the carbonyl oxygen of amide backbone in the membrane proteins (*e.g.*, KcsA (K channel of streptomyces A) through K^+^⋯O=C coordination bonding mode).^40^ The previously reported molecular mechanics calculations reveal that potassium ions display an average coordination number of 6.6 in KcsA membrane channel and 6.2 in bulk water.^40^ According to these information the pristine state was drawn and shown in Fig. 8, where K^+^ is represented as K^+^(H_2_O)_6._ This ion migrates in PVI film through breaking and forming of potassium ion-water molecule (K^+^⋯OH_2_) coordination bonds as shown in Fig. 8a (blue arrow). Whereas, the hydroxide ion migration occurs in long range *via* breaking and forming of hydrogen bonds with water molecules present in PVI–KOH film through the well-known Grotthuss-like mechanism.^41^ Since hydrogen bonding in water is a three dimensional network, we have shown only representative hydrogen bonds for clarity. Previous studies have shown that OH^−^ is a hypercoordinate species having four hydrogen bonds, HO^−^(H_2_O)_4_.^42^ Hence, we assumed that OH^−^ ion in pristine state is a hypercoordinate ion with three hydrogen bonds and one coordination bond with K^+^(H_2_O)_6_ ion (Fig. 8a) leading to form HO^−^(K^+^(H_2_O)_6_(H_2_O)_3_) species. In the charge separated state it transforms into a hypercoordinate hydroxide ion, HO^−^(H_2_O)_4_, as shown in charge separated state (Fig. 8b). The hydrogen bonding facilitates long range as well as fast HO^−^ ion migration in the PVI structure, which plays crucial role in determining charge separation and energy storage characteristics of the ITO/PVI–KOH/ITO planar MSC device.

## Conclusions

In summary, the ITO/PVI–KOH/ITO planar MSC devices with nanostructured PVI–KOH films are demonstrated on the EDLC formation mechanism and stability characteristics. The distribution of ionic charges and the corresponding electric double layer formation in the electrode/electrolyte interface were systematically studied using thin and relatively thick PVI–KOH in the planar device configuration. Single-cell of ITO/PVI–KOH/ITO planar MSC with a PVI–KOH thickness of ∼121 nm revealed higher volumetric capacitance (128 mF cm^−3^ at 10 mV s^−1^) and 92% capacitance retention (≤1400 cycles). But, the confinement of PVI–KOH thickness to the characteristic nanoscale (∼28 nm) remarkably enhanced the capacitance retention (∼95%) for more than 3500 continuous cycles. This significant deviation in EDLC characteristics contribute to the detailed understanding of the confinement effect of MSC devices not only for the use of EDL based energy storage applications but also for EDL based transistor applications.

## Experimental

### Materials and methods

#### Synthesis of poly(*N*-vinyl imidazole)

1-Vinylimidazole, azoisobuty–ronitrile (AIBN) and toluene were used as received from spectrochem chemical, India. In a typical reaction, 1-vinyl imidazole (0.1 mol) was mixed with toluene (40 mL) in a two neck round bottom flask fitted with a reflux condenser. Then, AIBN as initiator was subsequently added into the above reaction mixture. The reaction mixture was kept at constant stirring at 70 °C for 4 h under nitrogen atmosphere. The resultant polymer was washed several times with acetone. Then, the polymer was dried at 40 °C under vacuum for 6 h. The synthesized PVI was further confirmed using NMR analysis. Fig. S7 (ESI†) represents the ^1^H NMR spectrum of the prepared PVI. The ^1^H NMR signals for PVI is observed between the range 8 ppm and 1 ppm. The broad peaks observed between 6.6–7.4 ppm correspond to the imidazole protons. The broad peaks centred at 2.0 ppm and 3.0 ppm are ascribed to the polymer backbone of CH_2_ and CH groups, respectively.^32^

### Fabrication of solid polymer electrolyte-based flexible planar MSC

Poly(*N*-vinyl imidazole) (PVI)-based planar structure of ITO/PVI–KOH/ITO devices was fabricated using ITO-coated (thickness ∼130 nm) on flexible PET substrates. For planar device, two opposing ITO with a gap distance between ITO electrodes of ∼3 mm was carefully edged using diluted hydrochloric acid (HCl). Then, the edged ITO/PET substrate was cleaned with isopropyl alcohol and distilled water. To fabricate ITO/PVI–KOH/ITO planar MSC, homogeneous PVI–KOH solution was spin-coated on the substrate. The 0.1 g of PVI was dissolved in 5.0 mL of distilled water and then constantly stirred for 30 min at 90 °C to obtain a homogeneous solution. Separately, 50 wt% potassium hydroxide (KOH) salt was dissolved in 2.5 mL distilled water. After that, KOH solution was added into the homogeneous PVI solution. The resultant mixture of PVI–KOH was ultrasonicated for 15 min at room temperature. Finally, 150 μL of PVI–KOH homogeneous solution was spin-coated (Milman spin coater, India) on the ITO-edged PET substrate. The prepared PVI–KOH film thickness was found to be ∼121 nm using atomic force microscopy. Similarly, various thicknesses of the PVI–KOH films (∼66 nm, ∼28 nm) were prepared for planar MSCs by modifying the polymer to solvent ratio.

### Characterization

All the electrochemical characteristics of the planar MSC devices were performed with a two-electrode configuration. The cyclic voltammetry (CV), galvanostatic charge–discharge (GCD), electrochemical impedance spectroscopy (EIS) and open circuit potential (OCP) studies were carried out by Metrohm Autolab potentiostat/galvanostat instruments. The ionic conductivity (*σ*) of thin film was estimated using the following relationship,4
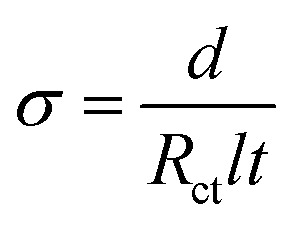
where, *d* is the distance between the ITO electrodes, *R*_ct_ is the resistance value obtained directly from EIS measurement, *l* is the length of the ITO electrode, and *t* is the film thickness.

The CV measurements were conducted with various scan rates (10 to 300 mV s^−1^) and specific potential window (0 to 1 V for single cell and 0 to 5.0 V for five series-cells). To study the GCD behavior, different charge–discharge current densities were used for charging/discharging of the planar MSC device. Furthermore, in GCD studies, two different voltage windows such as 0–1.0 V for single cell and 0–5.0 V for five series-cells were used. The CV curves were used to estimate the volumetric capacitance (*C*_V_) of the device, using the following relation,5
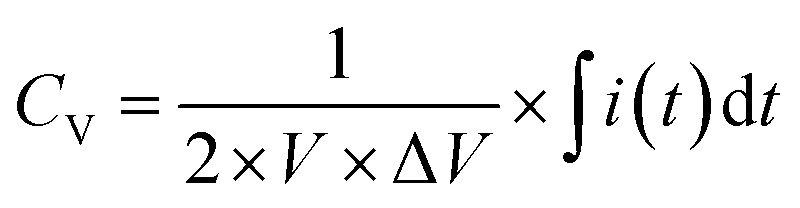
where, *I*(*t*) is the current measured during *C*_V_ testing, *t* is the time, *V* is volume of the device, and Δ*V* is potential range (=1.0 and 5.0 V for single and five-series cell planar MSC device, respectively).

The volumetric energy density (*E*_V_) and the power density (*P*_V_) of the planar MSCs were estimated from the CV plots for a scan rate in the range of 0.01–100 V s^−1^ by the following relations,6
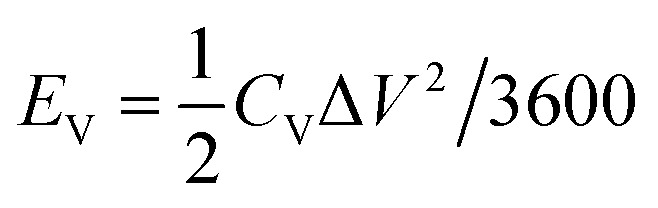

7 *P*_V_ = (*E*_V_/Δ*t*) × 3600

where, Δ*t* (in seconds) is the discharge time.

Also, the volumetric capacitance of planar MSC devices was estimated by GCD profiles using the relation8
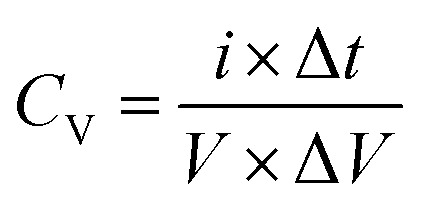
where, *i*, Δ*t*, *V*, and Δ*V* are the current, discharge time, volume of device, and potential window of the cell, respectively.

The prepared PVI polymer was characterized using X-ray diffractometer (XRD; X'Pert PRO, PANalytical, Cu Kα, *λ* = 0.15406 nm). Raman spectra for both pure PVI and PVI–KOH thin films were recorded using Laser Raman Microscope (Horiba Jobin Yvon-LabRAM HR Evolution). The FTIR study was done using FTIR spectrometer Bruker tensor 27. The polymerization process was further demonstrated using ^1^H NMR analysis using FT NMR spectrometer BrukerAvance III HD 400 MHZ. The surface morphology and thicknesses of the PVI–KOH films were observed using both field emission scanning electron microscope (FESEM) (Hitachi FE-SEM S-4800) and scanning probe microscope (Agilent technologies 5500 series) analysis.

## Conflicts of interest

There are no conflicts to declare.

## Supplementary Material

RA-010-D0RA08137J-s001

RA-010-D0RA08137J-s002
