# Supplementary material for: Ionic transport kinetics and enhanced energy storage in the electrode/poly(N-vinyl imidazole) interface for micro-supercapacitors
Source: RSC Adv. 2020 Dec 21;10(73):45019–27. doi: 10.1039/d0ra08137j (PMC9058607; doi:10.1039/d0ra08137j)
Supplement: RA-010-D0RA08137J-s002 [file RA-010-D0RA08137J-s002.pdf]

Electronic Supplementary Material (ESI) for RSC Advances.
This journal is © The Royal Society of Chemistry 2020

Electronic Supplementary Material (ESI) for RSC Advances

Ionic Transport Kinetics and Enhanced Energy Storage in the Electrode/Poly(N-vinyl imidazole) Interface for Micro-Supercapacitors

Karthik Krishnan,*[a] Selvakumar Karuthapandi,*[b] and Saranyan Vijayaraghavan[a]

[a] *Corrosion and Material Protection Division, CSIR-Central Electrochemical Research Institute, Karaikudi, Tamilnadu 630-003, India.*

[b] *Department of Chemistry, School of Science and Languages, VIT-AP University, Amaravati, Andhra Pradesh 522-237, India.*

E-mail: karthikk@cecri.res.in; selvakumar.k@vitap.ac.in

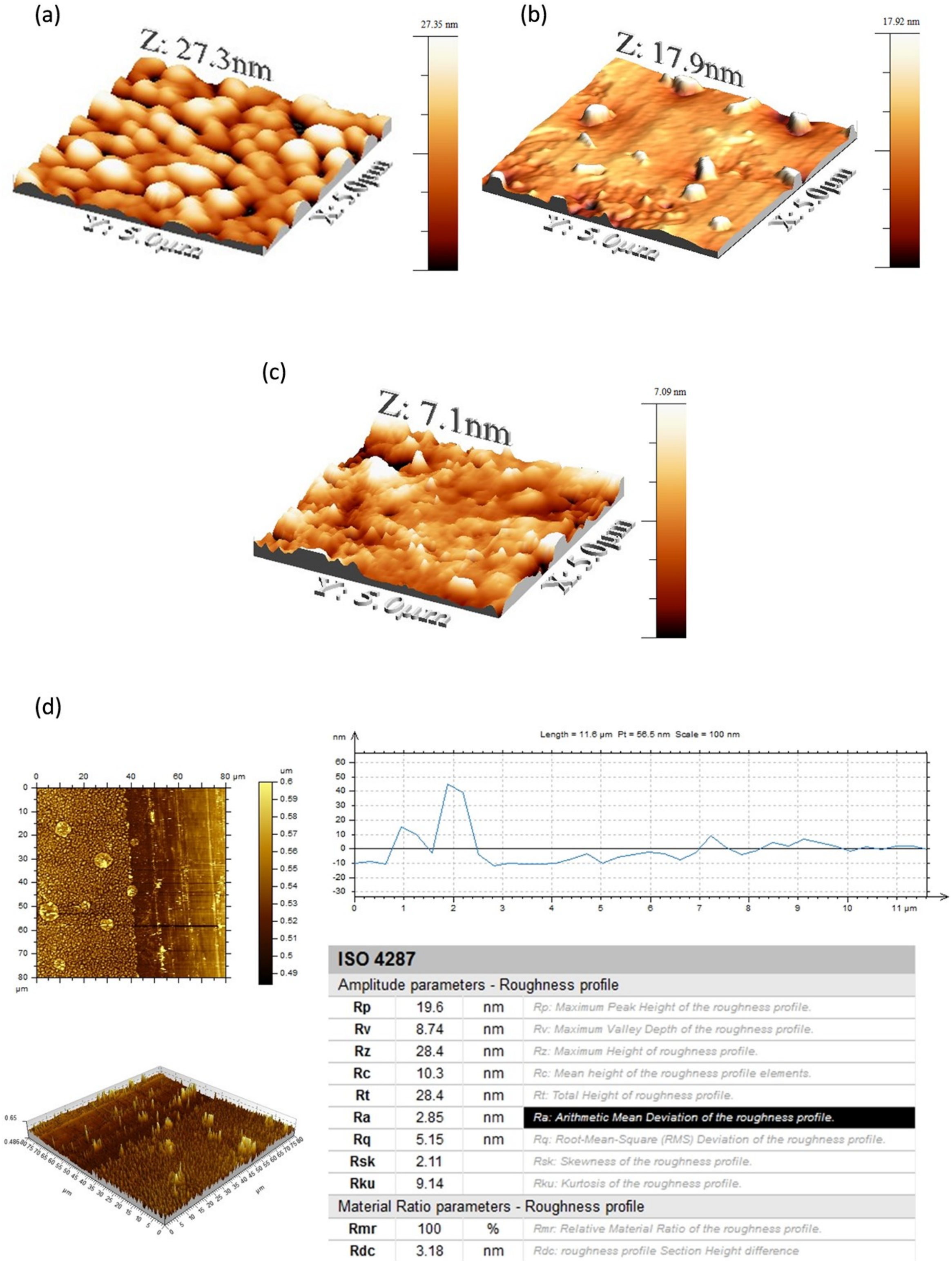

Fig. S1 3D AFM images of (a) ~121 nm, (b) ~66 nm, (c) ~28 nm, and (d) AFM measurement for thickness of the film (~28 nm) and the corresponding surface profiles.

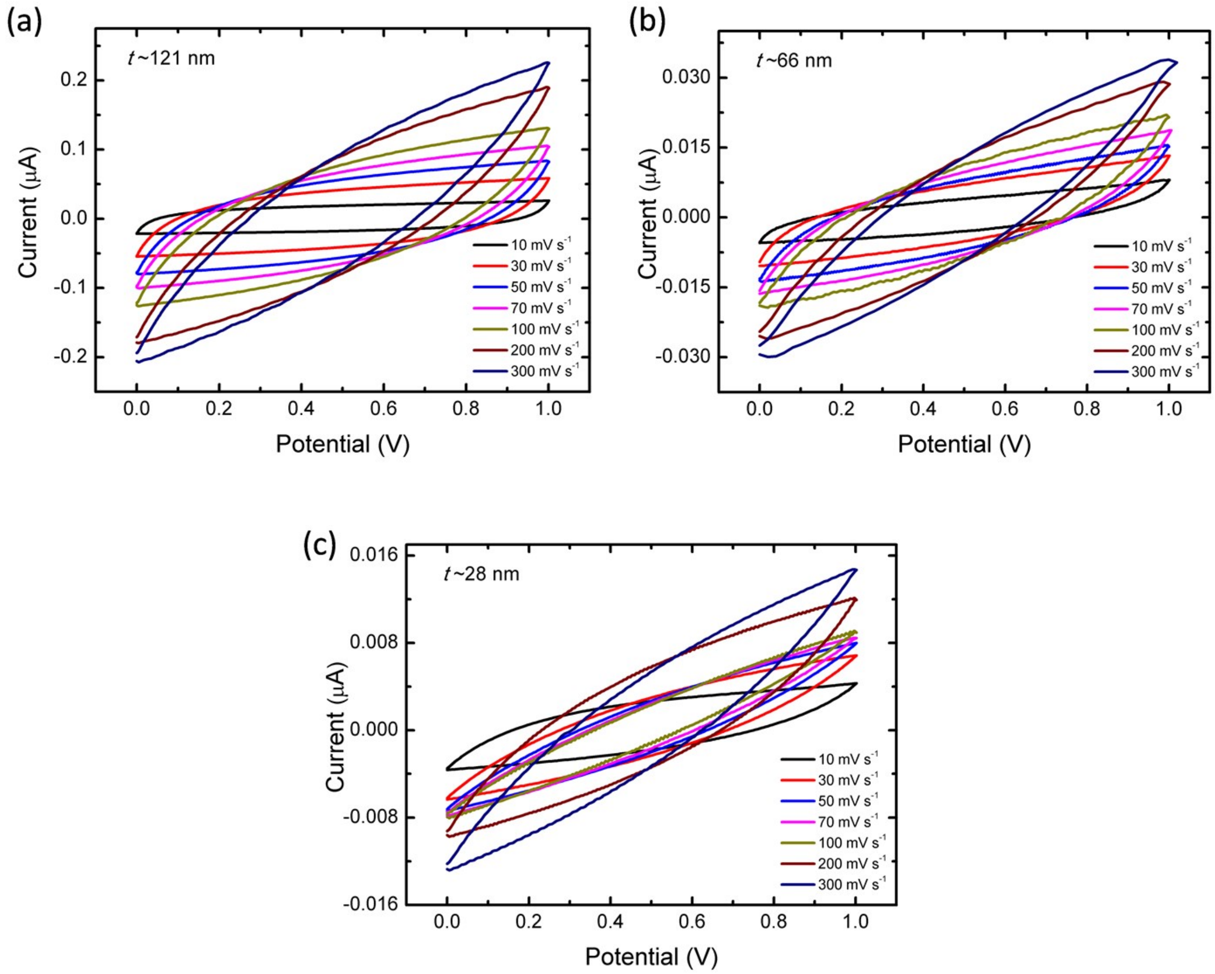

Fig. S2 Scan rate dependent CV curves of ITO/PVI-KOH/ITO planar MSC using various PVI-KOH film thicknesses ($t \approx 28$ to 121 nm).

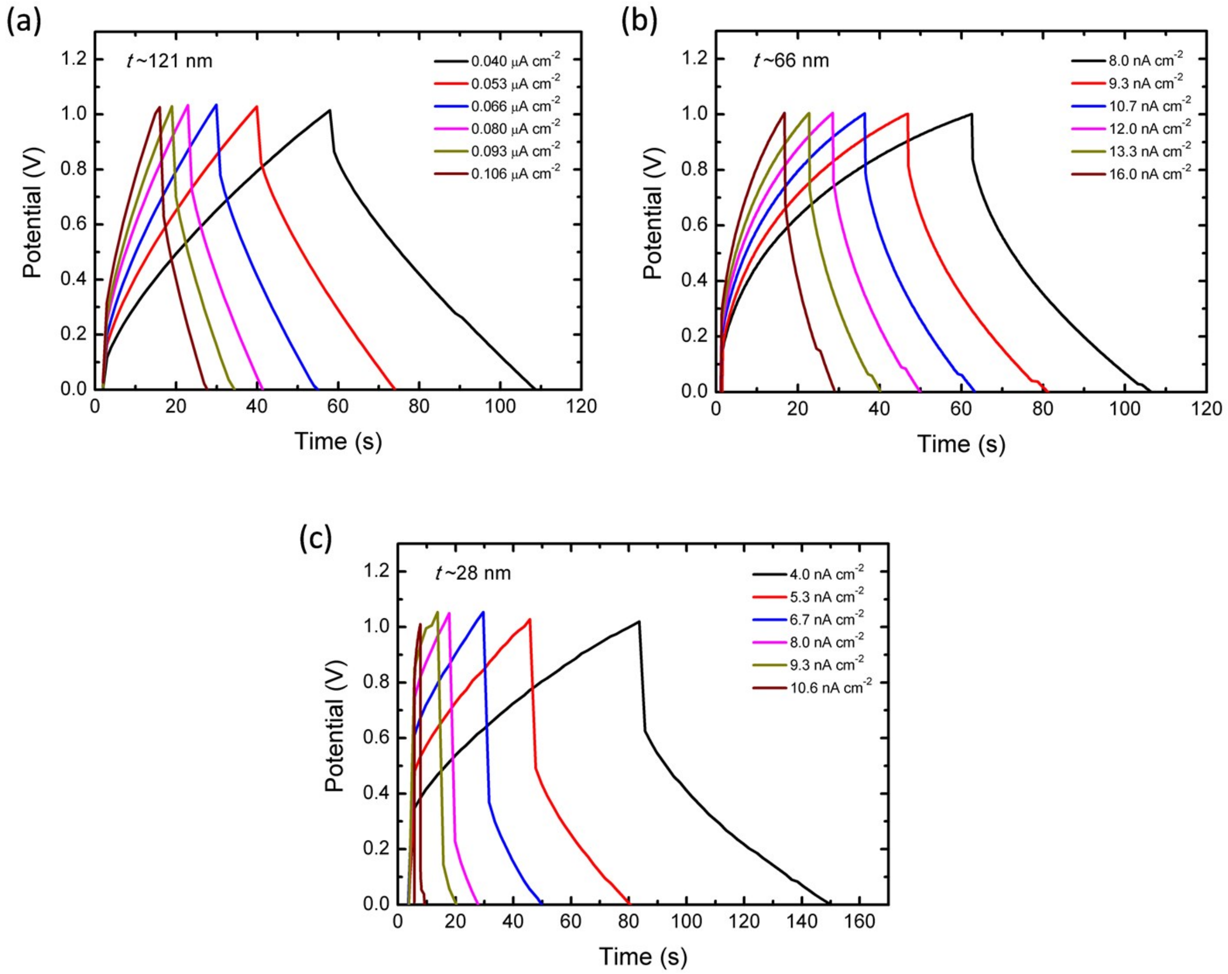

Fig. S3 PVI-KOH film thickness dependent charge–discharge profiles of the ITO/PVI-KOH/ITO planar MSC with various current densities.

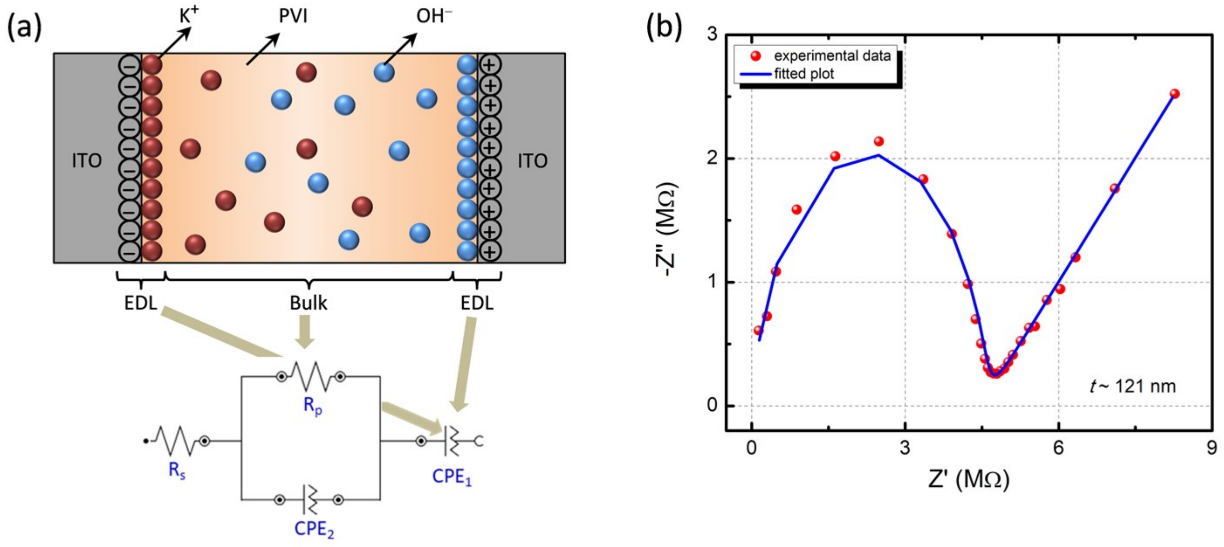

Fig. S4 (a) Equivalent circuit model describing the impedance behavior of the ITO/PVI-KOH/ITO planar MSC, and (b) the corresponding fitting plot of the device.

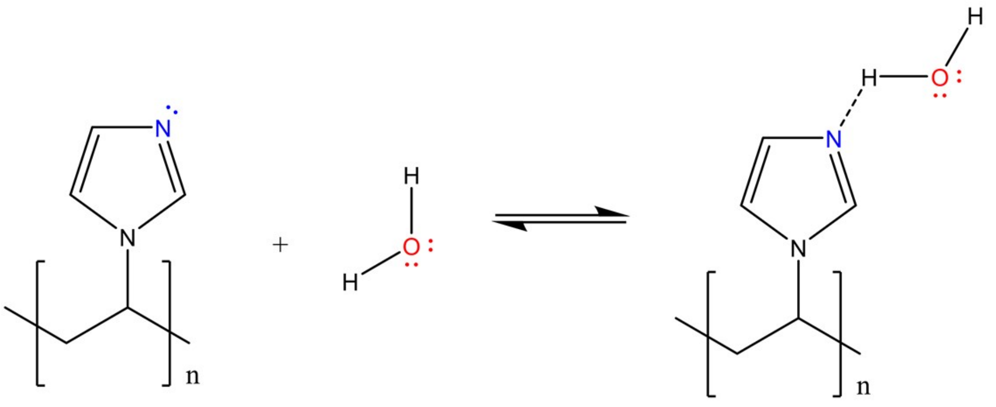

Fig. S5 Hydration of PVI through hydrogen bonding

Relative humidity (RH%) dependent ionic conductivity:

The ionic conductivity measurements were performed at room temperature and at various RH values. In the thin film, a two-probe method was used to obtain the conductivity data. The impedance data were obtained between the frequency range of 1 Hz and 1 MHz, with an applied potential of 50 mV. Atmospheres of various RH ranges were maintained by using different saturated salt solutions in their equilibrium states, including LiCl for ~11% RH, MgCl for ~32% RH, $Mg(NO_3)_2$ for ~51% RH, NaCl for ~75% RH, KCl for ~83% RH, KNO_3 for ~93% RH and K_2SO_4 for ~97% RH at room temperature (~30 °C).[1]

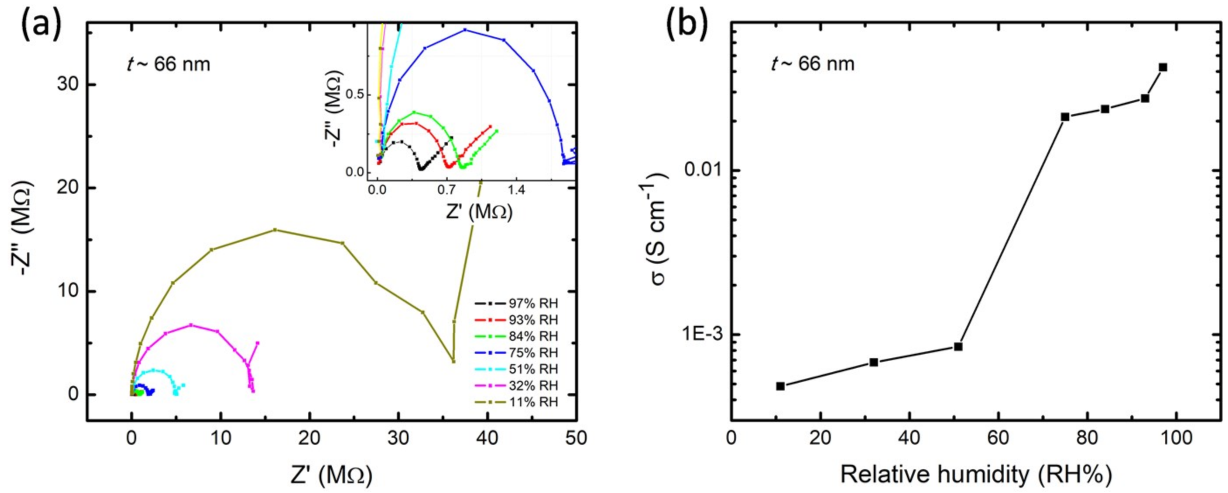

Fig. S6 (a) RH % dependent impedance plot, and (b) corresponding RH% versus ionic conductivity plot of PVI-KOH film (thickness (*t*) ~ 66 nm).

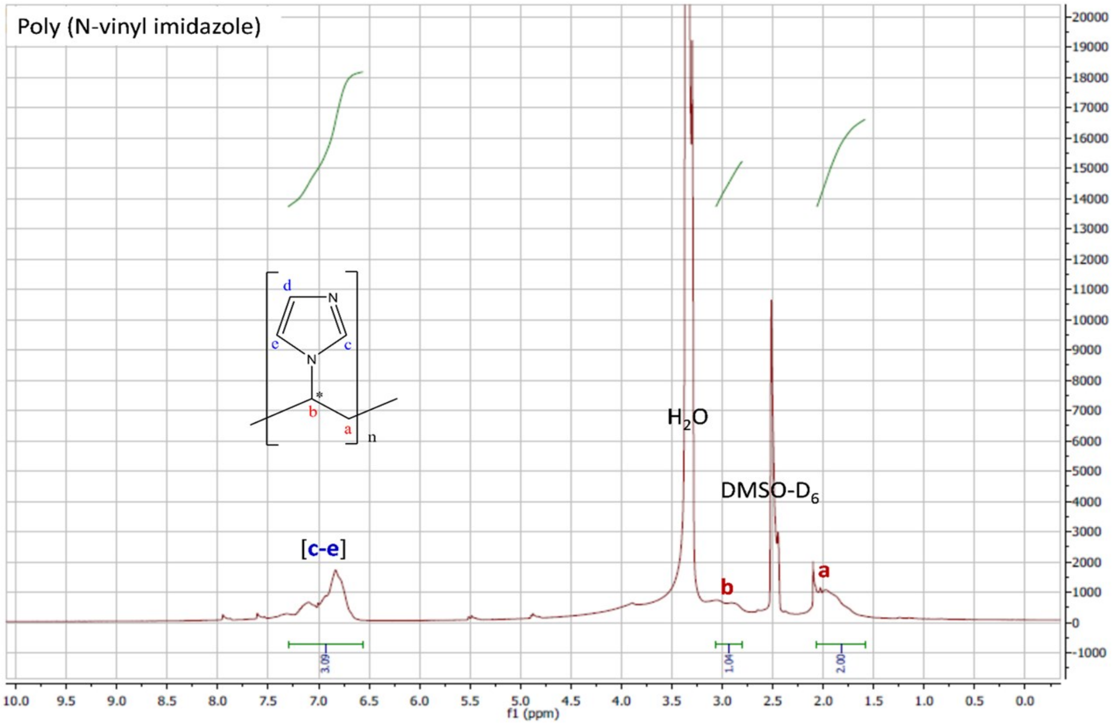

Fig. S7 ^{1}H NMR spectrum of the prepared PVI

Table S1. Electrical double layer capacitance characteristics are compared with the reported data.

Ref. No.	Electrode	Electrolyte	Capacitance (C)
[2]	Carbon-nanotubes	PVA-H_3PO_4	4.69 mF cm^{-2}
[3]	Heteroatom-doped porous graphene	PVA-H_2SO_4	1.36 mF cm^{-2} at 0.15 mA cm^{-2}
[4]	Direct laser write porous carbon	PVA-H_3PO_4	240 mF cm^{-3} at 10 mV s^{-1}
[5]	Boron-doped porous graphene	PVA-H^+	16.5 mF cm^{-2} at 0.05 mA cm^{-2}
[6]	RGMA ternary hybrid film	Ionic liquid gel	4.42 F cm^{-3} at 10 mV s^{-1}
[7]	Electrochemically reduced graphene oxide	25% KOH	0.48 mF cm^{-2} at 40 µA cm^{-2}
[8]	Transparent carbon film	PVA-H_3PO_4	409 mF cm^{-2} (branched CNC)
[9]	RGO-CNT (9:1)	3 M KCl	3 F cm^{-3}at 50 V/ s
[10]	MWCNT	PVA-H_3PO_4-H_2O &0.5 M H_2SO_4	1.8 mF cm^{-2}
[11]	Onion-like carbon	Et_4NBF_4/anhydrous propylene carbonate electrolyte	0.9 mF cm^{-2} at 100 V/s
This work	ITO	PVI-KOH	128 mF cm^{-3} at 10 mV s^{-1}

The GCD profiles shows the discharging effect of the planar MSC using multimeter as load, which can be seen in the real-time movies **ITO_PVI-KOH_ITO Planar MSC.mp4**.

References

1. D. Choudhury, J. K. Sahu and G. D. Sharma, *Ind. Crop. Prod.*, 2011, **33**, 211–216.

2. W. Yu, H. Zhou, B. Q. Li and S. Ding, *ACS Appl. Mater. Interfaces*, 2017, **9(5)**, 4597–4604

3. A. Basu, K. Roy, N. Sharma, S. Nandi, R. Vaidhyanathan, S. Rane, C. Rode and S. Ogale, *ACS Appl. Mater. Interfaces*, 2016, **8(46)**, 31841–31848.

4. J. B. In, B. Hsia, J. -H. Yoo, S. Hyun, C. Carraro, R. Maboudian and C. P. Grigoropoulos, *Carbon*, 2015, **83**, 144–151

5. Z. Peng, R. Ye, J. A. Mann, D. Zakhidov, Y. Li, P. R. Smalley, J. Lin and J. M. Tour, *ACS Nano*, 2015, **9(6)**, 5868–5875.

6. W. Liu, C. Lu, X. Wang, R. Y. Tay and B. K. Tay, ACS Nano, 2015, **9**, 1528–1542.

7. K. Sheng, Y. Sun, C. Li, W. Yuan and G. Shi, *Sci. Rep.*, 2012, **2**, 247.

8. H. Y. Jung, M. B. Karimi, M. G. Hahm, P. M. Ajayan and Y. J. Jung, *Sci. Rep.*, 2012, **2**, 773.

9. M. Beidaghi and C. Wang, *Adv. Funct. Mater.*, 2012, **22**, 4501–4510.

10. T. M. Dinh, D. Pech, M. Brunet and A. Achour, *J. Phys.: Conf. Ser.*, 2013, **476**, 012106.

11. D. Pech, M. Brunet, H. Durou, P. Huang, V. Mochalin, Y. Gogotsi, P. -L. Taberna and P. Simon, *Nat. Nanotechnol.*, 2010, **5**, 651–654.
